# Metabolic Engineered Biocatalyst: A Solution for PLA Based Problems

**DOI:** 10.1155/2018/1963024

**Published:** 2018-09-13

**Authors:** Sundus Riaz, Nosheen Fatima, Ahmed Rasheed, Mehvish Riaz, Faiza Anwar, Yamna Khatoon

**Affiliations:** ^1^Department of Biomedical Engineering and Sciences, National University of Sciences & Technology, Islamabad, Pakistan; ^2^Pakistan Agricultural Research Council, FQSRI, SARC, Karachi, Pakistan; ^3^PhD. Scholar, Sun Yat-Sen University (East Campus), Higher Education Mega Centre North, Guangzhou, China; ^4^MPH, London South Bank University, UK; ^5^Postgraduate Scholar, Department of Agriculture and Agribusiness Management, University of Karachi, Karachi, Pakistan

## Abstract

Polylactic acid (PLA) is a biodegradable thermoplastic polyester. In 2010, PLA became the second highest consumed bioplastic in the world due to its wide application. Conventionally, PLA is produced by direct condensation of lactic acid monomer and ring opening polymerization of lactide, resulting in lower molecular weight and lesser strength of polymer. Furthermore, conventional methods of PLA production require a catalyst which makes it inappropriate for biomedical applications. Newer method utilizes metabolic engineering of microorganism for direct production of PLA through fermentation which produces good quality and high molecular weight and yield as compared to conventional methods. PLA is used as decomposing packaging material, sheet casting, medical implants in the form of screw, plate, and rod pin, etc. The main focus of the review is to highlight the synthesis of PLA by various polymerization methods that mainly include metabolic engineering fermentation as well as salient biomedical applications of PLA.

## 1. Introduction

Polylactic acid (PLA) is a rigid thermoplastic polymer that has semicrystalline or amorphous geometry, depending on the optical purity of the polymer backbone [1]. Lactic acid has two optically active forms out of which L-lactic acid is the natural and most common form, whereas D-lactic acid is produced either by microorganisms or racemization. Furthermore, it acts much like comonomers which optimize the kinetics of crystallization for specific fabrication processes and applications [1]. Properties of PLA are similar to polyethylene terephthalate (PET) and polypropylene; these are petrochemical based polymer used for packaging applications [1]. PLA is a polymer which has wide range of applications in both biomedical and packaging industry, because it has ability to be stress crystallized, impact modified, filled, thermally crystallized, copolymerized, and processed in most polymer processing equipment [1]. It is unique in many ways and behaves like PET but also performs a lot like polypropylene. PLA has better organoleptic characteristics which makes it excellent for food contact and their related packaging applications [1].

Polylactic acid is produced by polymerization of lactic acid, and that is produced by two methods, i.e., chemical method and fermentation method [2]. Chemical method utilizes petrochemical resources followed by addition of HCN and specific catalyst to synthesized lactic acid [2]. On the other hand, fermentation method utilizes renewable resources, such as carbohydrate (monosaccharide and disaccharides) in the fermented broth to obtain lactic acid [3]. Optical purity of lactic acid is very important and hence is of major concern in production of PLA. Chemical method produces racemic mixture of both D (-) and L (+) lactic acid while fermentation method produces only one optically pure form of D (-) or L (+) lactic acid, respectively [2].

The main advantage of PLA that has encouraged its use in packaging industry is its high strength, biodegradability, antimicrobial, and antioxidant properties [3]. Although there is still a big market of petrochemical based polymer, these polymers have many disadvantages, because they adversely affect oil and gas resources which make them harmful as far as environment is concerned [3]. PLA is environmentally friendly because of its biodegradable properties and it can rapidly be degraded into less toxic byproducts like CO_2_ and H_2_O which saves environment from hazardous effects [3].

## 2. Synthesis of Lactic Acid Monomer for PLA Production

The cost factor for synthesis of lactic acid is raw material which is used in fermentation medium [4]. Production of lactic acid by fastidious lactic acid bacteria is usually a costly procedure [4]. Raw materials for lactic acid production are usually based on cheap polymeric waste and side stream materials [4]. These cheap materials are widely studied for high yield lactic acid [4]. For quality production of PLA, both optically and chemically pure lactic acid are required. Lactic acid synthesized from microbial strains produces optical pure lactic acid under optimized fermentation conditions [4].

**Figure 1:**
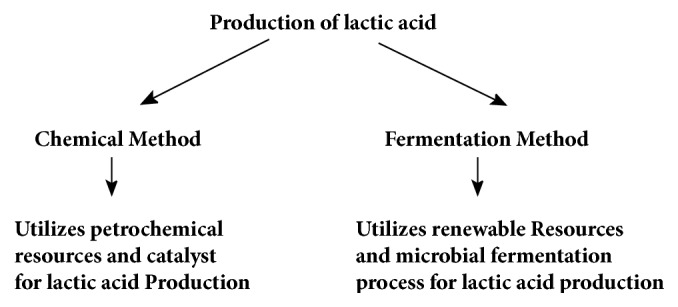
Methods of production of lactic acid.

Lactic acid yield from fermentation of monosaccharide usually has a very high molecular weight (> 90 %) [4]. Main impurity in the fermentation medium is being the cell mass itself that can be easily separated from the product [4].  Figure 1 illustrates the methods by which lactic acid is produced.

## 3. Chemical Method of Lactic Acid Production

**Figure 2:**
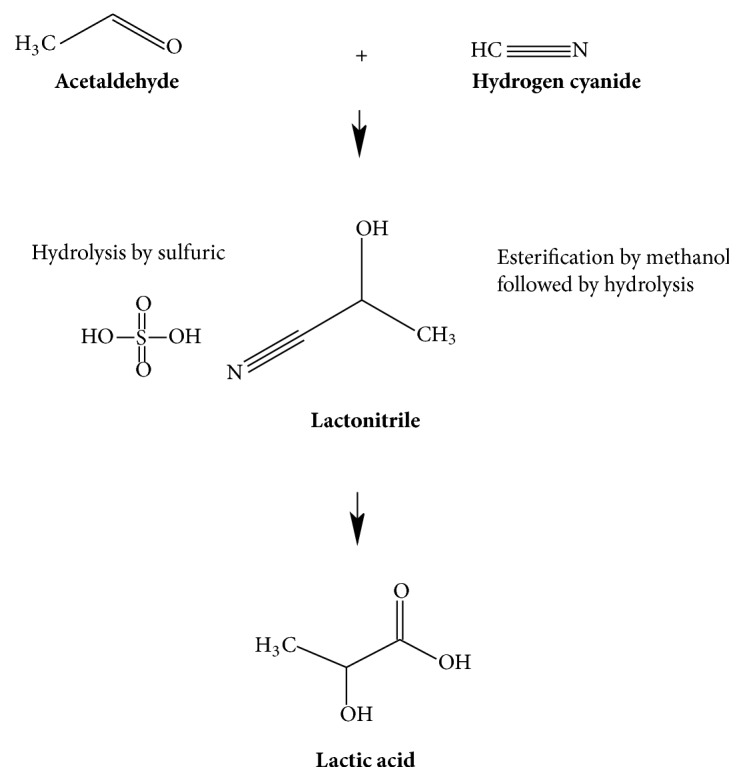
A schematic presentation of production of Lactic Acid by chemical process.

This method utilizes acetaldehyde reaction with hydrogen cyanide in the presence of catalyst to produce lactonitrile [19]. This reaction occurs in liquid phase and at high atmospheric temperature [19]. After completion of reaction, lactonitrile is purified and hydrolyzed to produce lactic acid [19]. This method produces racemic mixture of both D (-) and L (+) lactic acid [2]. Furthermore, the metal catalyst employed in this process is difficult to remove which makes it unfit in many applications.  Figure 2 presents the schematic illustration of chemical method.

## 4. Fermentation Method of Lactic Acid Production

Fermentation is an energy yielding process and is a characteristic of anaerobic bacteria [19]. Bacteria produce lactic acid by utilizing simple sugars like glucose, lactose, and galactose, without any requirement of heating process [19]. There are three types of fermentation process: (1) batch fermentation, (2) fed batch fermentation, and (3) continuous fermentation [19]. Batch and fed batch fermentation produce high concentration of lactic acid, whereas continuous fermentation produces higher productivity. Fermentation is usually carried out in controlled temperature and pH condition [19].

Both bacteria and fungi can produce lactic acid through fermentation but the yield of lactic acid by fungi is very low as compared to yield of lactic acid through bacteria [19]. To decrease cost for lactic acid production by fermentation, cheap raw material like lignocellulosic biomass is employed which is a promising feedstock due to its great availability, sustainability, and low cost as compared to refined sugars [20]. But the commercial use of lignocellulose for lactic acid production is still problematic because extensive pretreatment of enzyme is required to obtain fermentable sugars from lignocelluloses biomass [20].

Microorganism chosen for production of lactic acid should have high yield factor along with low cell mass at the expense of low cost raw material in low pH and at high temperature, along with negligible byproducts [21]. Continual improvements have been carried out in production and purification of lactic acid and are summarized in Table 1 [1].

## 5. Role of Bacterial Cultures in Lactic Acid Production

Lactic acid producing bacteria serve as starting material for production of lactic acid [3]. Both bacteria and fungi produce lactic acid but, for fungal production of lactic acid, aerial condition is required because it also shows low reaction rate [3].

Bacteria that carry out fermentation are divided into two groups, namely, homofermentative and heterofermentative, respectively. A homofermentative bacterium produces one product only at one time so production of side products can be minimized [3]. Example of such lactic acid bacteria by homofermentation are* Lactococcus*,* Enterococcus*,* Streptococcus*, and some* Lactobacilli* [4]. Homofermentative lactic acid bacteria metabolize hexose sugar entirely by Embden-Meyerhof pathway [4]. Industries are using homofermentative procedure for L-lactic acid production through specie of* Lactobacillus* genus, specifically with* Lactobacillus delbrueckii, L. amylophilus, L. bulgaricus, and L. leichmannii* [22]. Other than lactic acid bacteria, there are two other bacteria that produce lactic acid by fermentation. Thermotolerant* B. coagulans* utilizes glucose and xylose to produce yield of 96 % and 88 %, respectively; this is achieved at R_P_ (2.5 g/h) and product concentration (100 g/l) [4]. Yeast-like* Candida utilis *has been metabolically engineered by pyruvate decarboxylase deletion and L-lactate dehydrogenase expression to produce lactic acid from glucose with yield of 95% [4].

A heterofermentative bacterium produces ethanol and CO_2_ along with lactic acid [3]. Examples of organism that are heterofermentative are* Leuconostoc, Weissella,* and* Lactobacillus brevis *[4]. Heterofermentative bacteria utilize both hexose and pentose sugars to produce lactic acid. Heterofermentative bacteria are also employed to produce polyols such as mannitol, erythritol, ethanol, and acetic acid [4]. Recently,* Lactobacillus *strains are being utilized in fermentation for production of lactic acid [3].

New biotechnological improvements have been carried out so as to increase the yield of lactic acid production and to reduce side products. Metabolic engineering has been carried out in* Lactobacillus *strains so as to increase flux of lactic acid production [4]. In some metabolic engineering experiments, overexpression of genes does not cause increase in lactic acid yield. Such experiments are performed on* Lactobacillus plantarum* and* L. lactis*.* Lactobacillus plantarum* are metabolically engineered by overexpression of L-LDH but still show no increase in yield of lactic acid [4]. Similarly, overexpression of glyceraldehyde-3-P dehydrogenase (GAPDH) in* L. lactis* strain does not limit the glycolytic flux either in growing or resting cells [4].

**Figure 3:**
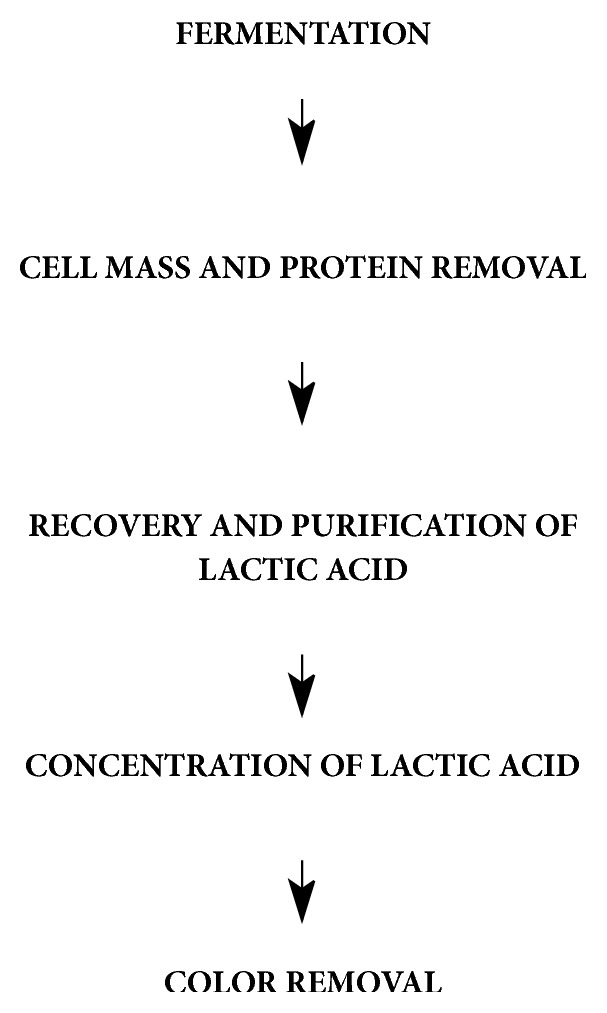
A schematic presentation of steps involved in production and purification of lactic acid by fermentation.

Metabolic engineering is also performed to obtain optically pure lactic acid. For example, altering activity of L-LDH in* Lactobacillus helveticus* is used for production of optically pure L-lactic acid [4].  Figure 3 shows steps involved in purification of lactic acid by fermentation.

## 6. Isolation and Purification of Lactic Acid from Fermentation Medium

**Figure 4:**
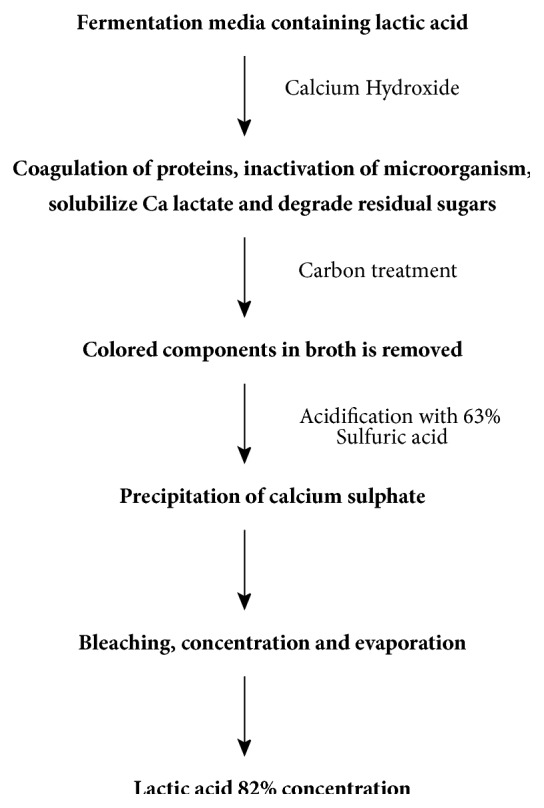
A schematic presentation of recovery and purification of lactic acid from fermentation of broth by adsorption.

For recovery and purification, lactic acid is adsorbed in suitable polymeric adsorbents [23]. Since polymeric adsorbents are nontoxic to fermentation broth, they can be used directly in fermentation medium [23]. In this process, strong alkali adsorbent is added which converts lactic acid into its basic salt [23]. The adsorbed lactic acid is then desorbed by adding strong acid like H_2_SO_4_ [23].  Figure 4 details the isolation scheme of lactic acid from medium.

Lactic acid can also be isolated from fermentation medium by reactive extraction [23]. This process requires liquid-liquid extraction along with reversible chemical complexion [23]. In this method, tertiary amine is used as extractant, L-decanol is used as diluent, and trimethylamine is used as stripping solution. Aqueous phase comprises fermentation medium [24]. To carry out extraction process, equal amounts of aqueous and organic phases are added and shaken for a definite period of time [24]. After shaking, aqueous phase is decanted and concentration of acid is determined on organic phase.

## 7. Production of Polylactic Acid

The following are two methods that are conventionally being used in polymerization of lactic acid.

- Direct polymerization [25]
- Ring opening polymerization of cyclic diester lactide [26]

### 7.1. Direct Polymerization

#### 7.1.1. Polycondensation of Lactic Acid

In this method, lactic acid (either produced chemically or by fermentation) is subjected to heat under vacuum at 50°C [25]. Disadvantage of this process is that it produces many side products of distillation which can contaminate the reaction mixture such as lactyl lactic acid [24]. Product of polycondensation yields low-molecular-weight polymer with low mechanical properties along with higher reaction times [26].

#### 7.1.2. Melt Condensation

This type of condensation is possible only if the temperature of the reaction remains above the melting temperature of the polymer [27]. This process produces high molecular weight polymer in short period of time. Reaction time for melt polymerization is ≤15 h. This method is cost-effective because of its simplified procedure but it requires sensitive reaction condition. To avoid these limitations, melt/solid polycondensation technique is developed that uses a binary catalyst which is tin dichloride hydrate and p-toluene sulfonic acid. Process involves thermal oligocondensates of lactic acid which were first subjected to melt polycondensation and later to solid state polycondensation [26]. So, after reaction, high molecular weight PLA is obtained having molecular weight around 600,000. Melt condensation is a relatively economical and easy to control process. Melt condensation is affected by factors such as temperature, reaction time catalyst, and pressure [27]. So consideration must be employed to these factors to obtain high molecular weight PLA [27]. Metal catalyst used in this process is difficult to remove and makes resulting polymer unfit for biomedical applications.

### 7.2. Ring Opening Polymerization of Cyclic Diester Lactide

#### 7.2.1. Formation of Lactide

Lactide is oligomer of polylactic acid and for its formation lactic acid is added to a reactor containing vacuum and a stirrer, and zinc oxide or Sn(OEt)_2_ is added in to the reaction mixture as catalyst [26]. As water is removed at high temperature the oligomerization is promoted; after that, temperature is quickly increased and yellow liquid is distilled which on cooling converts into needle-like crystals. These crystals are recrystallized at least 4 times to obtain pure colorless crystals of lactide [26].

#### 7.2.2. Ring Opening Polymerization

Ring opening polymerization (ROP) requires reaction initiator (Tetraphenyltin) for polymerization [28]. In this method, pure lactide is placed in clean, dried polymerization tube; the appropriate amount of initiator is dissolved in benzene and placed in polymerization tube [28]. The whole system is kept in oil bath under vacuum or nitrogen at 60-100°C. The reaction mixture is allowed to sublime [28]. Following sublimation, the tube is again immersed in oil bath, and after predetermined interval the contents of polymerization are removed and kept at -15°C. The extent of polymerization is determined by gel permeation chromatography [28]. Disadvantage of ROP processes is that it requires high temperature which initiates side reactions that hinder its propagation [15]. ROP is divided into two categories, namely, cationic ring opening polymerization and anionic ring opening polymerization [15]. The process of cationic ROP can produce low-molecular-weight poly lactic acid while anionic ROP process can lead to racemization [15]. Furthermore, ROP uses tin as catalyst which is incorporated into polymer system, making the resulting polymer unfit for biomedical applications.

## 8. Bioproduction of Polylactic Acid

Polylactic acid and its copolymer can be prepared via genetic manipulation of microorganism following the process of fermentation [14]. Metabolically engineered E. coli with propionate CoA transferase and polyhydroxyalkanoate (PHA) synthase can be used for production of polylactic acid and its copolymers [14]. This metabolically engineered bacterium utilizes glucose as a substrate for the production of polylactic acid and its copolymers [14]. To enhance biosynthesis of polylactic acid and its copolymers, MBEL 6–19 PHA synthase (PhaC1Ps6–19) is engineered with in vitro mutagenesis to generate lactyl CoA, which enhance the PLA production, which is analyzed by gas chromatography [14]. Metabolic pathways of E. coli for production of PLA are further modified by deletion of genes, which are ackA, ppc, and adhE genes, respectively [16]. Promoters' genes are also replaced from ldhA and acs to trc promoter based on in silico genome-scale metabolic flux. Recombinant strain of E. coli can be used for making homopolymer lactic acid [16].

Chemical medium having pH 7.0 is used for production of recombinant strains of E. coli [17]. This medium contains potassium dihydrogen phosphate, ammonium phosphate, magnesium sulphate hepta hydrate, citric acid, and trace metal solution per liter, respectively [17]. Seed cultures of E. coli are prepared in Luria-Bertani medium. After incubation, seed culture is inoculated to MR medium containing glucose [17]. Flask cultures are kept under temperature of 30°C at rotary shaker.

**Figure 5:**
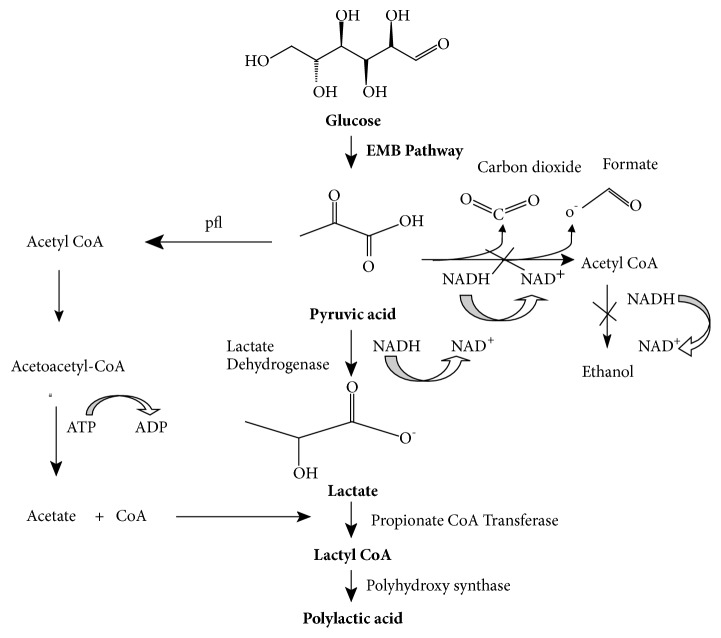
A schematic presentation of production of polylactic acid by metabolic engineering.

Polymer produced by bacteria is analyzed by gas chromatography, flame ionization detector [17]. To determine amount of PLA formed by fermentation, dried pellets of PLA are subjected to methanolysis with 15% sulphuric acid with internal standard as benzoic acid [17]. Products of methanolysis, such as carboxylic acid and lactate, are analyzed by gas chromatography. Methyl lactate was analyzed on mass spectrometric detector. For determination of molecular weights, gel permeation chromatography is used. PLA is subjected to differential scanning calorimetry (DSC) [17].  Figure 5 summarizes production of polylactic acid through metabolic engineering and Table 2 summarizes genetically manipulated microorganisms that are used in PLA production.

## 9. Source and Cloning of Gene Containing Propionate CoA Transferases

Propionate CoA transferase (PCT) gene is present in* Clostridium propionicum* which is regarded as alanine fermenting organism [29]. This organism is found in black mud of San Francisco bay [29]. Other organisms that produce Propionate CoA transferase are* Megasphaera elsdenii, Bacteroides ruminicola, *and* Clostridium homopropionicum* [29]. When PCT enzyme gene is overexpressed in E. coli, a serious metabolic disorder is observed, which causes death of all recombinant E. coli, when an inducer is added in an isopropyl-.beta.-D-thio-galactoside (IPTG)-inducible protein expression system having a T7 promoter [30]. Due to this, constitutive expression system which expresses gene weakly but continuously with growth of a microorganism is induced [30].

For cloning of Propionate CoA transferase gene, a degenerated primer pair is introduced [29]. This primer is used to amplify a 300-bp fragment of genomic DNA obtained from* C. propionicum* using PCR [29]. Labelled PCR product is used for screening a library of genomic DNA from* C. propionicum *using k-ZAP-Express phage vector [29].

## 10. Source and Cloning of Gene Containing PHA Synthase

Polyhydroxyalkanoates synthase enzyme is mainly found in most genera of bacterium and members of the family Halobacteriaceae of the Archaea [31]. This enzyme utilizes thioesters of hydroxyalkanoic acids as substrate and converts them into polyhydroxyalkanoic acids [31]. For cloning of PHA gene, 8 different strategies are employed [31]. Strategy A has enzymatic approach which is employed to screen clones for functional expression of PHA gene [31]. In strategy B, after transposon mutagenesis, homologous gene probes have been obtained [31]. This strategy is used to identify the respective gene intact within the same genome [31]. In strategy C a well characterized R. eutropha PHA synthase gene is used which is used to identify corresponding genes from genomic libraries [31]. Strategy D focuses on design of short oligonucleotides with short highly conserved stretches of PHA synthases [31]. In strategy E, PHA synthase protein is purified and their oligonucleotide is designed from its N-terminal. This strategy is used to identify gene from a genomic library [31]. The most successful strategy for cloning of PHA synthase is strategy F, in which genomic libraries are screened to obtain PHA-negative wild-type organism [31]. Strategy G aimed to clone heterologous phaC genes to a PhaC-negative mutated organism [31]. In strategy H, homologous proteins encoding PHA are cloned subsequently [31]. In strategy I, bacteria is allowed to grow in a medium without carbon for storing polymer [31]. Bacteria from which PHA genes are cloned are* Paracoccus denitrificans, Rhodobacter capsulatus, Chromobacterium violaceum, Pseudomonas putida* BM01,* Methylobacterium extorquens, Comamonas acidovorans, Ectothiorhodospira shaposhnikovi, Synechocystis* sp., and* Zoogloea ramigera,* respectively [31].

## 11. Deletion of Other Pathways

Red recombinase expression plasmid is constructed for one-step inactivation of gene encoding pyruvate formate lyase, fumarate reductase, and LacI transcriptional repressor, respectively [14]. This Recombinant E. coli containing Red recombinase expression plasmid was cultivated at 30°C [14]. The expression of Red recombinase is induced by adding 10 mM L-arabinose [14]. Pyruvate formate lyase gene is deleted by homologous recombination in two steps [14]. Firstly, 1234bp DNA fragment which contains lox71 site, chloramphenicol resistance gene, and lox66 site fused together to be obtained by PCR product [14]. Primers utilized are FDpflB1 and RDpflB1, respectively [12]. The final pcr product introduced to E. coli has pKD46 gene [14]. Screening of colonies is done on Luria-Bertani (LB) agar plate containing chloramphenicol and subsequently by direct colony PCR [14]. AdhE gene is deleted by using FDfrd1, RDfrd1, FDfrd2, and RDfrd2 for frdABCD, FDadhE1, RDadhE1, FDadhE2, and RDadhE2 primers, while lacI gene is deleted by FDlacI1, RDlacI1, FDlacI2, and RDlacI2 primers, respectively [14]. E. coli harboring chloramphenicol resistant mutants is transformed with pJW168 primer and ampicillin-resistant gene [14]. Screening is done on Luria-Bertani agar containing 100 gml^−1^ ampicillin and 1 mM IPTG. Positive colonies are cultivated and screed by PCR [14].

## 12. Biomedical Application of PLA

Polylactic acid is a group of bioresorbable polymers that show higher tensile strength and it erodes into harmless components when interacting with physiological fluids [32]. Due to its high tensile strength, it can be braided into sutures, stents, and scaffolds. PLA based biomaterials can be manufactured by injection molding, extrusion, spinning film, and casting process [18]. Rate of absorption of PLA depends upon molecular weight, morphology, and enantiomeric purity of PLA as PLA with high molecular weight is used to absorb it completely [18].

Nowadays, collagen and hyaluronan-based matrices are among the most popular scaffolds in clinical use, because their substrate is essential for cartilage support [18]. Furthermore PLA based scaffolds are being extensively used in tissue engineering [32].

PLA based drug delivery systems can be used in the form of pellets, nanoparticles, microcapsules, microparticles, and sustained release dosage forms. In addition, PLA based devices and drug delivery systems have been extensively used in tumors.

## 13. Conclusion

PLA is a biodegradable polymer used in manufacturing many biomedical devices and packaging applications. It either can be found as a sole polymer or can copolymerize with other polymers. PLA is produced from polymerization of lactic acid, and monomer lactic acid synthesized by chemical or fermentation method. Chemical method produces racemic mixture of both D(-) and L(+) forms of lactic acid while fermentation method only produces optically active L(+) form of lactic acid, which is required for PLA production. Lactic acid, whether produced from fermentation or chemical method, is further polymerized by two methods: direct polymerization of lactic acid and ring opening polymerization. These polymerization methods have many drawbacks. Firstly, the polymer synthesized from direct polymerization method produces low mechanical strength polymer; secondly, ring opening polymerization uses catalyst that makes polymer unsuitable for biomedical applications. Synthetic formation of lactic acid contains many limitations such as inability to form required L- lactic acid isomer and low product yield because it utilizes by-product as a reactant, thus making it a high cost procedure.

Recent methods of PLA utilize genetic manipulation of microorganisms that can produce polylactic acid directly by fermentation. In this context, E. coli seemed suitable for genetic modification with the insertion of propionate CoA transferase and polyhydroxyalkanoate (PHA) synthase gene that have ability to produce lactyl CoA and PLA directly by fermentation. Industries are using homofermentative procedure for L-lactic acid production because it leads to greater yield and lower amount of by‐products. The PLA produced by fermentation is found to be mechanically fit for biomedical applications along high molecular weight, strength, and yield as compared to conventional methods.

## Figures and Tables

**Table 1:** Microorganism along with their yield of production of lactic acid by fermentation.

**Sr. No.**	**Microorganism producing lactic acid**	**Substrate involved**	**Genetic modification**	**Yield of lactic acid**
1	*T. aotearoense* SCUT27[5]	Lignocellulosic biomass[5]	Engineered to block the acetic acid formation pathway[5]	0.93 g/g glucose with an optical purity of 99.3%[5]

2	*Lactobacillus amylovorus * ATCC 33622[6]	Liquefied corn starch[6]	Nil	20 g l^−1^ h^−1^[6]

3	*L. helveticus* [6]	Whey[6]	Nil	35 g l^−1^ h^−1^[6]

4	*Enterococcus faecalis *CBRD01[7]	Glucose[7]	Nil	5 g l−1 h−1[7]

5	*L. delbrueckii *NCIM 2025[8]	Cane molasses concentration of 150 g/L (equivalent to 78 g total sugar). [8]	*adh-ve *mutant by UV radiation[8]	78±1.2 (g/g) [8]

6	*L. plantarum *LMISM6 [9]	Molasses 193.50 g L-1[9]	NIL	94.8 g L-1[9]

7	Thermophilic *Bacillus* sp. XZL4[10]	Corn stover hydrolyzate 162.5 g L-1[10]	NIL	1.86 g L-1 h-1[10]

8	*Lactococcus lactis*[11]	Glucose 60 gl-1 [11]	Nil	35 gl-1[11]

9	Escherichia coliBAD-ldh[12]	1g l-1 of fructose[12]	Overexpression of L-ldh gene derivative[12]	0.62 g l-1[12]

10	Escherichia coli[13]	56 g/L of crude glycerol[13]	Overexpression of GlpK/GlpD gene[13]	50 g/L of L-lactic acid[13]

**Table 2:** Table summarizes genetically manipulated microorganisms used in PLA production.

**Sr. No.**	**Name of Microorganism**	**Genetic Manipulation**	**Substrate Utilized**	**Yield of PLA**	**Analytical Technique**
**1**	Escherichia coli[14]	Insertion of propionate CoA-transferase and polyhydroxyalkanoate (PHA) synthase gene[15]	Glucose [14]	43 wt% [14]	Gas Chromatography[14]

**2**	Escherichia coli[16]	Insertion of propionate CoA-transferase and polyhydroxyalkanoate (PHA) synthase gene [16] Knocking out the ackA, ppc, and adhE genes[16] Replacing the promoters of the ldhA and acs genes with the trc promoter[16]	56 wt% from glucose[16]	55-86 mol%[16]	Gas Chromatography[16]

**3.**	Escherichia coli[17]	Introduction of propionate CoA transferase (PctCp) gene from Clostridium propionicum And 19 polyhydroxyalkanoate (PHA) synthase 1 (PhaC1Ps6-19) from Pseudomonas sp. into Escherichia coli for the generation of lactyl-CoA endogenously and incorporation of lactyl-CoA[17]	62wt% glucose[17]	20–49 mol%[17]	Gas Chromatography[17]

**4.**	Escherichia coli[18]	introduction of heterologous pathways having engineered propionate CoA-transferase and polyhydroxyalkanoate (PHA) synthase in to E. coli for generation of lactyl-CoA [18]	46 wt% glucose[18]	70mol% [18]	Gas Chromatography[17]
